# The management of calcified teeth with iatrogenic errors with static-guided and freehand endodontic technique

**DOI:** 10.4317/jced.63480

**Published:** 2026-03-30

**Authors:** Afzal Ali, Anuja Ikhar, Ezgi Doğanay Yıldız, Hakan Arslan

**Affiliations:** 1 Department of Conservative Dentistry and Endodontics, Pacific Dental College and Hospital, Udaipur, Rajasthan, India; 2 Reader, Department of Conservative Dentistry and Endodontics Sharad Pawar Dental College and Hospital, India3; 3 Datta Meghe Institute of Higher Education and Research (DMIHER), Sawangi, Wardha, Maharashtra, India; 4 Department of Endodontics, Faculty of Dentistry, Bursa Uludag University, Bursa, Turkey; 5 Department of Endodontics, Faculty of Dentistry, Medeniyet University, İstanbul, Turkey

## Abstract

**Background:**

The endodontic management of teeth with pulp canal calcification (PCC) is challenging and may result in iatrogenic errors in the form of excessive tooth structure loss and perforation. The present case reports aims to describe the management of calcified teeth with iatrogenic canal deviation using static-guided (SG) and conventional free hand endodontic technique.

**Case Presentation:**

A 23-year-old male patient referred to the clinic with the chief complaint of pain over the upper incisor teeth region. Periapical radiography and CBCT scan revealed pulp canal calcification (PCC) of maxillary central incisors (#11 and #21), and periapical radiolucency in relation to #21 and #22. The patient was informed about the treatment options including SG endodontic approach and the course of treatment was determined based on the patients' decisions. The endodontic treatment of teeth #21 and #22 was initiated with conventional free hand technique. After a perforation on the labial root wall, the patient opted for saving the tooth (#21) using SG endodontic technique. With the help of CBCT, intra-oral Scanner and a 3D printer, a guide was fabricated. The root canal was negotiated and the endodontic treatment was completed. Similarly, the endodontic treatment of a 21-year-old female patient with iatrogenic apical deviation was performed using the same endodontic protocol, except for the use of a static-guide. Follow-up examination revealed clinically asymptomatic patients with the radiographic signs of periapical healing in both the cases.

**Conclusions:**

Tooth substance loss during access cavity preparation plays a vital role in determining the prognosis of the tooth. The teeth with PCC are categorized as cases with a high difficulty level which are often prone to iatrogenic errors. SG endodontic technique is a safe approach and provides predictable outcome in the management of cases with high difficulty level.

## Introduction

Pulp canal calcification (PCC) can be defined as dentinal hard tissue deposition within the root canal system (1). PCC is often encountered in teeth with history of trauma. Dentinal hard tissue can also be deposited due to aging or in response to various stimuli such as caries, restorative procedures, or orthodontic tooth movement (1 , 2). The presence of PCC alone does not constitute an indication for the endodontic treatment. However, when accompanied by pulpal and/or periapical pathologies, endodontic treatment is indicated (3 , 4). Endodontic treatment of such teeth is challenging and time-consuming because of the difficulties of negotiating the calcified root canals (5). The detection of the root canal might only be achieved by removing significant amount of peri-cervical dentin which has a significant effect on the fracture resistance of the tooth (6). There is a relatively high risk of encountering complications such as inaccessibility of the root canal, perforation, and file separation (7). Kvinnsland et al.(8) reported 20% of the perforations in their study on endodontic management of teeth with calcified root canals. In order to shorten the chair-side time and reduce the possibilities of complications, guided endodontic treatment has emerged as an alternative to conventional freehand technique. It is more reliable method for accessing the teeth with PCC (9). Static-guided (SG) endodontic technique employs digital planning and three-dimensional (3D) printing to create a customized template for accessing the root canal system. A cone beam computed tomography (CBCT) scan is superimposed over intraoral stereolithography (STL) data. Access path is virtually planned using 3D planning software. This ensures minimal dentin removal and accurate canal localization. The purpose of these case reports are to present the clinical outcome of SG and a conventional freehand approach for the endodontic treatment of calcified teeth with an iatrogenic error.

## Case Report

**Figure 1:**
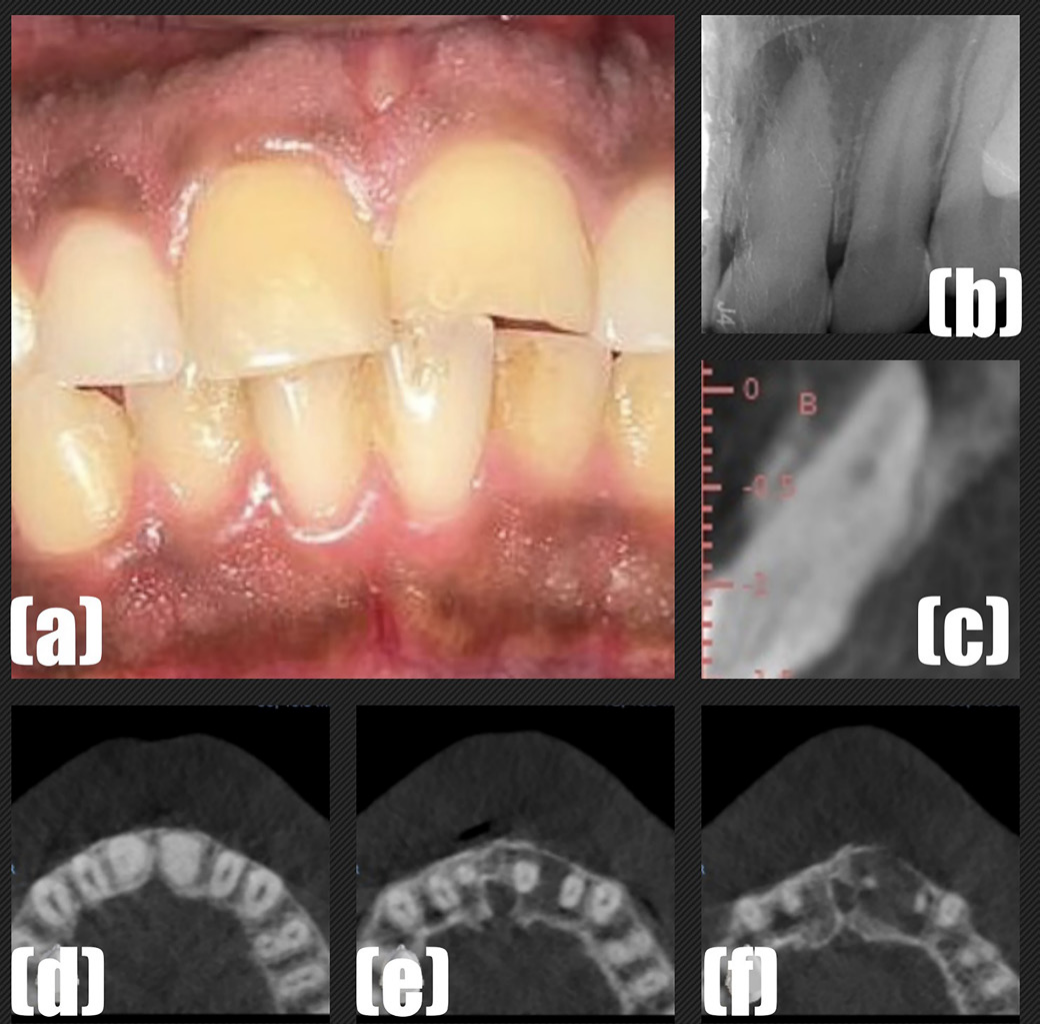
(a) Clinical aspect of teeth. (b) The periapical radiography which revealed PCC of #21 tooth and periapical radiolucency in relation to teeth #21 and #22. (c-e) CBCT showing PCC of teeth #11 and #21.

Case 1: Iatrogenic perforation with conventional technique and its management using the SG approach: A 23-year-old male patient was referred to the Department of Conservative Dentistry and Endodontics with a chief complaint of pain in upper incisor teeth region. There was a history of trauma in the childhood. Clinical examination: Discoloration of teeth #11 and #21 was noticed (Fig. 1a).

1

**Figure 2:**
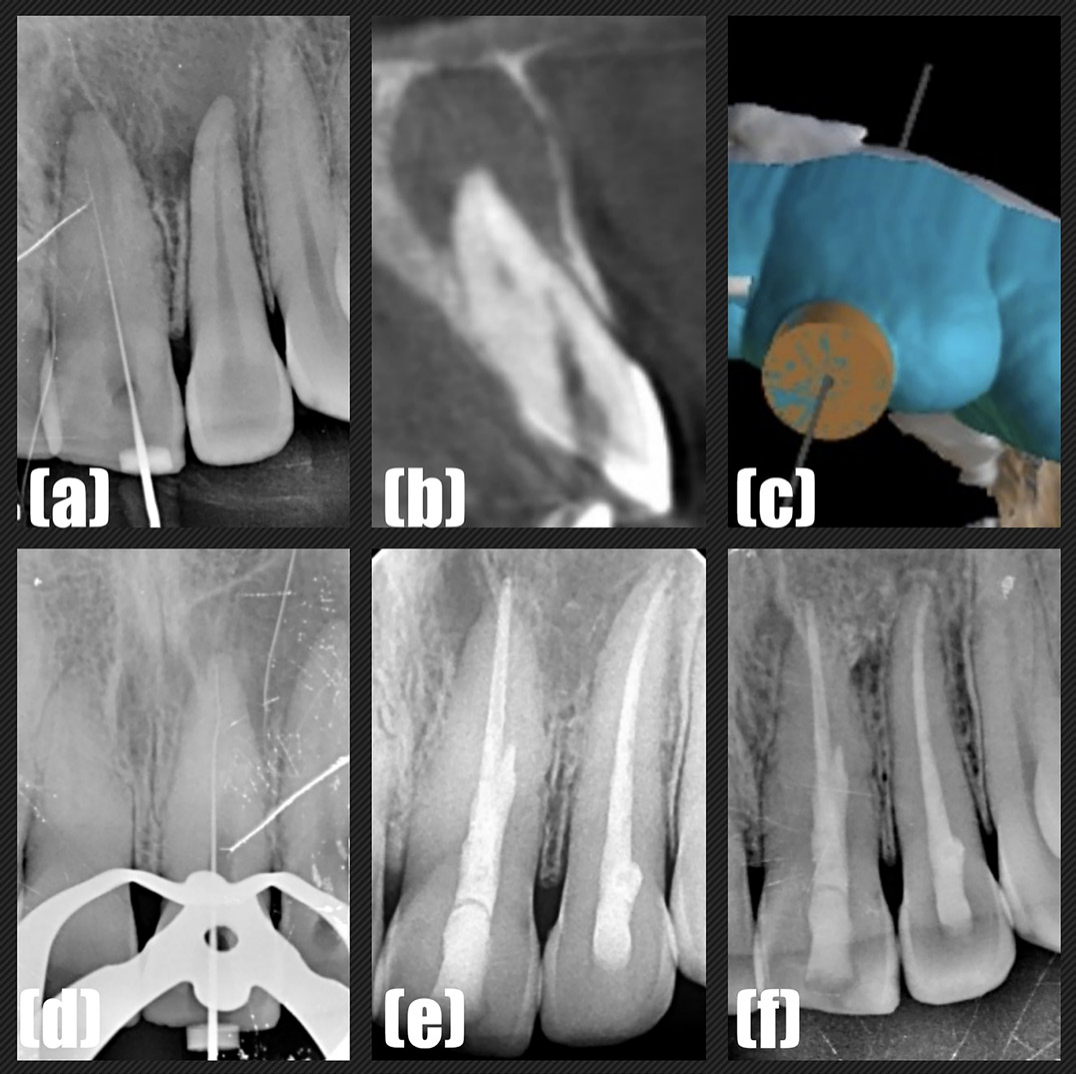
(a) The periapical radiography showing the presence of perforation. (b) CBCT showing the presence of perforation on the labial root wall. (c) The guide designed and 3D printed. (d) The periapical radiography which confirmed the working length. (e) Postoperative periapical radiograph. (f) Follow-up radiograph one year after the completion of the root canal treatment.

Tooth #21 was tender to percussion and palpation. Pulp sensibility test (cold test and electrical pulp test) was performed on the upper incisor teeth (#11, #12, #21 and #22) and tooth #11 was responsive while the teeth #21 and #22 were non-responsive. Radiographic Assessment (2D and 3D): Periapical radiograph revealed PCC of teeth #11 and #21 and periapical radiolucency in relation to teeth #21 and #22 (Fig. 1b). A CBCT (GE Healthcare, USA) scan was performed with a limited field of view (FOV), 100-micron voxel size, and with exposure parameters of 84 kV, 6.0 mA, and 12 s. CBCT images confirmed the PCC of teeth #11 and #21 (Fig. 1c-f). In addition, a radiolucent area was seen in the periapical region of teeth #21 and #22. Owing to the challenges associated in teeth with PCC, tooth #11 was kept under observation, as it was asymptomatic, while endodontic treatment of teeth #21 and # 22 was planned. Teeth #21 and #22 were diagnosed with pulp necrosis and symptomatic apical periodontitis. The risks and potential complications during root canal treatment were explained to the patient and the course of treatment was determined based on the patients' decisions. Thus, the endodontic treatment of teeth #21 and #22 was initiated with the conventional free hand technique. First visit: After the administration of local anesthesia, the root canal treatment of tooth #22 was initiated under rubber dam isolation. The working length was determined using an Ai-Pex apex locator (Woodpecker, Guilin, China). The root canal was prepared using Ni-Ti rotary instruments (Neolix EDMax, Châtres-la-Forêt, France) till size 40/.04. Calcium hydroxide intracanal medicament was placed in tooth #22. During the access cavity preparation for tooth #21, rubber dam was not applied in order to observe the axis of the tooth. An access cavity was prepared under magnification (3x dental loupes; Eighteeth Medical, Changzhou, China). After a punch cut in the enamel using a round bur, troughing was performed using a micro endodontic bur (Endoguide EG5; SS White, Lakewood, USA) and a Mueller bur. A micro endodontic bur has an overall length of 34 mm, a head length of 1.5 mm, and a tip diameter of 0.28 mm. Multiple periapical radiographs were taken during the procedure to locate and negotiate the calcified canal. The root canal could not be located/negotiated and the patient was recalled after 2 days. Second visit: Troughing was continued for tooth #21 using the same burs and monitored with multiple radiographs during the procedure. Bleeding was seen in the root canal, and the root perforation was suspected. Periapical radiograph (Fig. 2a) confirmed the presence of root perforation.

2

**Figure 3:**
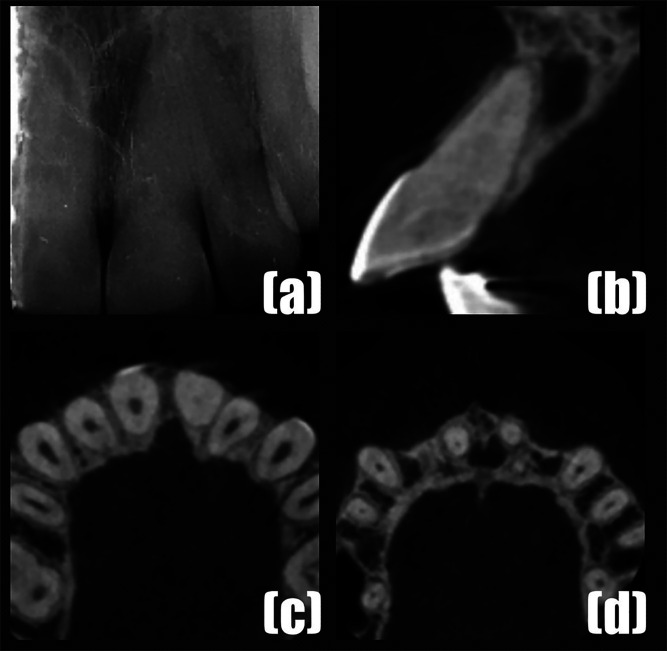
(a) The periapical radiography which revealed PCC and periapical radiolucency in relation to teeth #21. (b-d) CBCT showing PCC of tooth #21.

3D Guide designing: After the explanation of the iatrogenic complication (perforation), the patient desired for SG endodontic technique. Hence, the second CBCT (GE Healthcare, USA) scan was performed (Fig. 2b). Intraoral STL file of the maxillary arch was obtained using the AutoScan-DS200 Dental 3D Scanner (Shining 3S Tech Co. Ltd., Hangzhou, China) and the CBCT data was superimposed on it. An ideal access path was planned virtually to locate the root canal using software (exocad Asia Ltd., Tsim Sha Tsui Kowloon, Hong Kong) for teeth #11 and #21 (Fig. 2c) and printed using 3D printer (Formlabs Inc., Somerville, MA, USA) and SG resin (Formlabs Inc.). Tooth #21 was treated with the SG approach, while #11 was kept under follow-up as it was asymptomatic and responsive to pulp sensibility tests. Third visit: The guide was placed on the teeth and checked for its fit. A micro endodontic bur was used in order to prepare the path through the guide (Endoguide EG5 and a Mueller bur). After the negotiation of the root canal, the tooth was isolated using a rubber dam (Hygenic, Coltene Whaledent Inc., Akron, OH, USA). The working length was determined using an electronic apex locator (Ai-Pex) and confirmed by periapical radiograph (Fig. 2d). The root canal preparation was done using Neolix Edmax files up to size 40.04 and enlarged further to size #60 K file as the canal was already drilled with the burs. rrigation was performed. The root canal and the perforation defect were filled with MTA (Proroot MTA Dentsply) and the access cavity was restored with resin composite for tooth #21 (Fig. 2e). The endodontic treatment of tooth #22 was completed with the conventional approach. The patient was recalled after one year following the root canal treatment. Clinical examination revealed absence of tenderness to percussion or palpation. Radiographic examination revealed resolution of periapical radiolucency in relation to teeth #21 and #22 (Fig. 2f). Case 2: Apical deviation during conventional freehand endodontic approach and its follow-up A 21-year-old female patient reported with the chief complaint of tooth pain over the upper front tooth region. The patient had a history of trauma in the childhood. Clinical examination: Tooth #21 was tender to percussion and palpation. Pulp sensibility test (cold test and electrical pulp test) was performed in the upper incisor teeth (#11, #21) and tooth #21 was non-responsive. Radiographic Assessment (2D and 3D): The periapical radiograph suggested PCC of tooth #21 (Fig. 3a).

3

**Figure 4:**
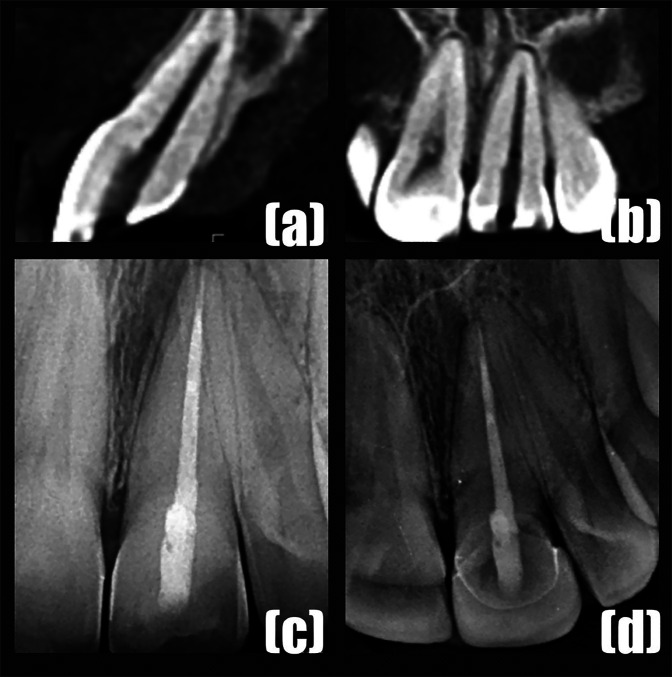
(a-b) CBCT showing the presence of apical deviation. (b) Postoperative periapical radiograph. (d) Follow-up radiograph Two year after the completion of the root canal treatment.

A CBCT (GE Healthcare, USA) scan was performed using similar exposure parameters as for Case 1 to assess and confirm degree of PCC (Fig. 3b-d). The #21 tooth was diagnosed with pulp necrosis; symptomatic apical periodontitis. With the informed consent, the conventional endodontic treatment was initiated at the request of the patient. The endodontic procedure was performed by the same operator (A.A.) using the same treatment protocol as described in Case 1 except for the use of an endodontic guide. First visit: Access cavity was prepared and an attempt to locate the root canal was performed. After a punch cut in the enamel using a round bur, troughing was performed using a micro endodontic (EG5 and Mueller) bur. Periapical radiographs were taken between attempts. As the root canal could not be negotiated, the patient was recalled after 3 days. Second visit: Troughing was continued using the same burs and monitored with multiple periapical radiographs. Apical deviation was suspected and was confirmed with the CBCT scan (Fig. 4a,b).

4

As the patient insisted on the non-guided approach, the canal was transported apically and prepared till size #60 K-file. The root canal was filled with apical plug of MTA and backfilled using GP and Endoseal MTA sealer (Maruchi S korea). The access cavity was restored with composite resin (Fig. 4c). The patient was asymptomatic in the follow-up examination after 2-years (Fig. 4d). Volumetric tissue loss: In both cases, excessive tooth substance loss was calculated using the CBCT scan that was taken after encountering the complications. Tooth substance loss other than the original canal path were identified in post-complication CBCT data, and the volume estimation were performed for these regions. To achieve this, a 3D reconstruction of the tooth (from pre-op and post-op CBCT data) was exported to an STL format using a 3D slicer (version 5.0). A python script was written especially for this case. The script automatically imports the pre-op and post-op STL files, calculates their respective volumes, and then outputs the difference in mm3. This python script used a python module called pydicom (version 3.0.1). In Case 1, the volumetric loss was estimated to be 5.23 mm3. In case 2, the loss caused by apical deviation was 1.11 mm3.

## Discussion

PCC is usually a late-term sequelae following dental trauma. The literature has reported varying degrees of calcifications in teeth following trauma ranging from 4 to 24%. In the present cases, the patients had a history of a fall in childhood, and hence trauma is the probable cause for the PCC and periapical changes. In Case 1, PCC and the discoloration of teeth #11 and #21 was observed. Although tooth #21 was treated, the endodontic procedure was not performed on tooth #11, since no sign of pulpal and/or periapical disease was evident (3 , 4). In Case 2, the tooth #21 was tender to percussion with radiographic signs of periapical changes. According to the American Association of Endodontists (AAE), the Endodontic Case Difficulty Assessment Form and Guidelines categorizes indistinct canal paths as cases displaying a "high" level of difficulty (10). In such cases, the risk of complications such as excessive tooth substance loss, file separation or perforation, may negatively affect the prognosis of such teeth (6 , 7). Guided endodontic treatment is an alternative to conventional free hand technique to reduce these risks, improve prognosis and decrease the chair-side time (9). In Case 1, the patient did not accept the guided endodontic treatment earlier, although the risks were explained. In Case 2, the patient insisted on a conventional endodontic approach, even after encountering the apical deviation. According to the AAE Endodontic Case Difficulty Assessment Form and Guidelines, previous complications during access to root canal (such as a non-negotiated canal, ledge, perforation or separated instrument) are categorized as having a "high" difficulty level (10). Similarly, cases with calcifications are also considered to be highly challenging (10). CBCT is recommended for the management of such cases (11). The endodontic guide requires CBCT data and intra-oral scan. Therefore, preoperative and intra-operative CBCT were taken in the present case reports. However, a small FOV was used to ensure the dose reduction. In the present cases, the occurrence of perforation resulting during the conventional free hand endodontic access cavity preparation made the case more complicated. After the complication was explained to the patient, the patient in Case 1 accepted SG endodontic treatment. Thus, the selection of treatment modality was based on patient decision. There are case reports in the literature showing the successful outcome with guided endodontic approach in teeth with PCC (12 , 13). However, there are few case reports on the guided approach for the management of calcified teeth with iatrogenic perforations (14 , 15). Loureiro et al. presented the management of a case with labial root perforation in the middle third of a central incisor using SG endodontic technique. The root canal was located using guided endodontics and 6-month follow-up showed successful results, both clinically and radiographically. Casadei et al. presented the management of a case with lateral perforation in the apical third of an upper second premolar tooth using SG and a one-year follow-up showed successful results both clinically and radiographically. In these case reports, as in the present case report, the successful management of complications with guided approach can be observed. Vision enhancement with magnification provides a more detailed view of root canal intricacies (16). Bowers et al. (17) compared the effects of operating microscope magnification (8x), dental loupe magnification (2.5x), and unaided vision on fine motor skills when used in endodontics. They reported that the use of 2.5x loupes resulted in significantly better performance compared to unaided vision. The use of an operating microscope provided a significant increase in accuracy scores compared to both unaided vision and 2.5x loupes. In the present case report, a 3x dental loupe was used for magnification during the procedure. The use of an operating microscope during the management of the present cases could have facilitated the identification of the canals by providing better magnification and, consequently, improved visualization. This is a limitation of the present case report. Although guided endodontic treatment requires time for its planning and guide fabrication. However, the shortening of the patient's chair side time is an advantage for both the patient and the clinician (18). Connert et al. (19) reported that the use of a static-guide in the treatment of teeth with canal calcification preserves more tooth structure and may therefore contribute to the long-term survival of the tooth. The present case reports did not compare the techniques in terms of the amount of the tooth substance loss. However, in cases with complications such as perforation or apical deviation, the amount of tissue removed may be of more critical importance. In this context, the use of a guide could be particularly valuable. The advantages of using static guides such as increased rapidity and predictability made it an important aid in the treatment of challenging cases. However, the cost and availability of inter-occlusal space are the influential parameters in clinical decision-making.

## Conclusions

The present case report described the management of calcified teeth with an iatrogenic error using SG endodontic technique and a conventional freehand approach. The SG technique is a safe method which provides predictable results with regard to the management of complex cases (Case 1).

## Data Availability

All data generated or analyzed during this case report are included in this published article.
